# Symptomatic Treatment of Vascular Cognitive Impairment (STREAM-VCI): Protocol for a Cross-Over Trial

**DOI:** 10.2196/resprot.9192

**Published:** 2018-03-20

**Authors:** Jolien Fleur Leijenaar, Geert Jan Groeneveld, Wiesje Maria van der Flier, Philip Scheltens, Erica Surya Klaassen, Henry Chanoch Weinstein, Geert Jan Biessels, Frederik Barkhof, Niels Daniël Prins

**Affiliations:** ^1^ Alzheimer Center & Department of Neurology Amsterdam Neuroscience VU University Medical Center Amsterdam Netherlands; ^2^ Centre for Human Drug Research Leiden Netherlands; ^3^ Department of Epidemiology & Biostatistics VU University Medical Center Amsterdam Netherlands; ^4^ Department of Neurology Onze Lieve Vrouwe Gasthuis West Amsterdam Netherlands; ^5^ Department of Neurology Brain Center Rudolf Magnus University Medical Center Utrecht Utrecht Netherlands; ^6^ Department of Radiology and Nuclear Medicine Amsterdam Neuroscience VU University Medical Center Amsterdam Netherlands; ^7^ Institutes of Neurology and Healthcare Engineering University College London London United Kingdom; ^8^ Brain Research Center Amsterdam Netherlands

**Keywords:** Vascular cognitive impairment, dementia, methylphenidate, galantamine, cognition, Magnetic Resonance Imaging, small vessel disease, vascular disease, clinical trial

## Abstract

**Background:**

People with vascular cognitive impairment (VCI) constitute a clinically heterogeneous group, but previous symptomatic drug trials in VCI did not take this clinical heterogeneity into account. Executive dysfunction and memory impairment are the cognitive domains that are most frequently impaired in VCI, and these impairments are likely to reflect vascular damage to specific neurotransmitter systems, which opens the possibility for targeted symptomatic treatment directed at specific neurotransmitters.

**Objective:**

Here we describe the design of the “Symptomatic Treatment of Vascular Cognitive Impairment” (STREAM-VCI) trial. In this proof-of-concept study, we investigate whether people with VCI with executive dysfunction due to vascular damage to the monoaminergic neurotransmitter system differentially respond to a monoaminergic challenge, whereas people with VCI with memory dysfunction associated with vascular damage to the cholinergic system will in turn respond to a cholinergic challenge.

**Methods:**

The STREAM-VCI is a single center, double blind, three-way cross-over trial among 30 people with VCI, in which subjects received a single dose of galantamine, methylphenidate, or placebo on separate occasions. The most important inclusion criteria were a diagnosis of VCI with a Mini-Mental State Examination score of ≥16 and a Clinical Dementia Rating of 0.5-1.0. For each person, the challenges consisted of a single 16 mg dose of galantamine, 10 mg of methylphenidate, and placebo, in random order on three separate visits. Change in performance in executive functioning and memory was assessed directly after the challenge using standardized neuropsychological tests. We will correlate a positive response to the cholinergic and monoaminergic treatment with differences in structural and functional connectivity at baseline using structural magnetic resonance imaging (MRI), diffusion tension MRI, and resting-state functional MRI.

**Results:**

The protocol of this study is approved by the Medical Ethics Committee of VU University Medical Center and the competent authority. The first participant was enrolled in April 2014. In September 2017, enrolment for the study was completed. We expect to publish the results in 2018.

**Conclusions:**

STREAM-VCI is the first study to investigate the association of a response to a cholinergic and monoaminergic treatment with structural and functional connectivity of the monoaminergic and/or cholinergic systems on MRI. We aim to predict on an individual basis which individuals show a positive response to a cholinergic and/or monoaminergic challenge in people with VCI. This may be instrumental in moving in the direction of individually-tailored pharmacological interventions based on MRI measures in people with VCI.

**Trial Registration:**

ClinicalTrials.gov NCT02098824; https://clinicaltrials.gov/ct2/show/NCT02098824 (Archived by WebCite at http://www.webcitation.org/6xhO7Ya1q)

## Introduction

The two most prevalent cognitive symptoms in people with vascular cognitive impairment (VCI) are executive dysfunction and memory impairment [1,2]. However, the presence and extent of these symptoms varies largely between people with VCI. Previous intervention studies did not take this into account, and until now, there is no approved symptomatic treatment for people with VCI.

Recent insights in the neuropharmacological basis of cognitive symptoms in VCI suggest that executive dysfunction is largely related to dysfunction of the monoaminergic systems (noradrenergic and dopaminergic) that project mainly from the locus coeruleus. Memory impairment is thought to be related to dysfunction of the cholinergic system projecting form the nucleus basalis of Meynert [3-5]. Also, neuronal networks, such as the default mode network (DMN), is assumed to be involved in attention, concentration, and executive function. Vascular damage is thought to cause impairment of the cholinergic and monoaminergic neurotransmitter systems by damaging specific white matter tracts and cause disturbances in the neuronal networks such as the DMN [6-8], resulting in cholinergic and/or monoaminergic deficits.

Galantamine is a drug that increases the availability of acetylcholine in the synaptic cleft and previous studies have shown positive results on memory in people with probable Alzheimer’s disease [9-14]. Executive functioning might be improved by increasing norepinephrine and dopamine transmitters with methylphenidate. This drug can increase the concentrations of dopamine and norepinephrine in the synaptic cleft [15-17]. Two previous studies have shown a slight improvement on cognition, based on Mini-Mental State Examination (MMSE) scores, in people with dementia following methylphenidate use [18,19].

Here we describe the design of the trial “Symptomatic Treatment of Vascular Cognitive Impairment” (STREAM-VCI). In this proof-of-concept study, we aim to study the individual change on performance on executive function and/or memory function after a single dose of methylphenidate and galantamine, compared to placebo, in people with VCI. We will correlate the change on performance after the pharmacological challenge with functional and structural connectivity of the damaged monoaminergic and cholinergic neurotransmitter systems using a structural magnetic resonance imaging (MRI) and resting-state functional magnetic resonance imaging (rs-fMRI) [20-24]. Based on this information we aim to understand and predict which individuals will benefit from a certain pharmacological treatment. This could be a step forward towards personalized drug treatment based on MRI measures.

## Methods

### Study Design

The STREAM-VCI is a single center, double-blind, three-way, case cross-over pharmacological challenge study, in which participants received a single dose of galantamine, methylphenidate, or placebo on separate occasions. Participants were primarily recruited from the Alzheimer Center of the VU University Medical Center (VUmc). Also, subjects were recruited after referral from the Department of Neurology of the Utrecht University Medical Center (Utrecht) and the outpatient clinic of the following hospitals: Groene Hart Ziekenhuis (Gouda), Spaarne Gasthuis (Haarlem), and Tergooi (Blaricum). Subjects were enrolled between April 2014 and September 2017. The trial is registered at the clinical trial register: NCT02098824.

### Subjects

Subjects were people with VCI ranging from vascular mild cognitive impairment to vascular dementia, according to the definitions of the American Heart Association/American Stroke Association [25]. Eligible people who satisfied the inclusion and exclusion criteria were selected (Textbox 1). Individuals fitting the inclusion and exclusion criteria were given the study information and at least one week’s time to consider participation in the study. The enrolment of 30 subjects was complete in September 2017.

### Randomization

Eligible subjects who fulfilled the inclusion and exclusion criteria were given the study medication in a randomized order. Latin squares balanced for first-order carry-over effects were used, called Williams squares. Because of the uneven number of treatments, a pair of squares was required to ensure balance for first-order carry-over effects. Randomization was carried out by an independent researcher. Medication was identified by project and protocol number, packing number, expiration date, storage requirement, and contents.

**Inclusion and exclusion criteria for Symptomatic Treatment of Vascular Cognitive Impairment.**

Inclusion criteria

- Outpatients
- Objective executive dysfunction and/or memory impairment on neuropsychological tests
- Imaging evidence of cerebrovascular disease (white matter changes (Fazekas ≥2), (lacunar) infarcts, and/or (micro)hemorrhages)
- Mini-Mental State Examination score of ≥16
- Clinical Dementia Rating of 0.5-1
- No contraindication for treatment with a cholinesterase inhibitor or methylphenidate
- Assessed by the treating neurologist as mentally capable of understanding the implications of study participation
- Presence of an informant/caregiver at the information visit and signing of informed consent

Exclusion Criteria

- Clinically-relevant history of abnormal physical or mental health interfering with the study as determined by medical history taking and physical examinations obtained during the screening visit and/or at the study day as judged by the investigator
- Clinically-relevant abnormal laboratory results, electrocardiogram (ECG) and vital signs, or physical findings at screening and/or at the start of the study day (as judged by the investigator)
- Unwilling or unable to stop smoking on the study day until the end of the study day
- Other causes that can explain cognitive symptoms including but not limited to: delirium, multiple sclerosis, amyotrophic lateral sclerosis, progressive supranuclear palsy, mental retardation, infectious encephalitis that led to persistent cognitive deficits or head trauma with loss of consciousness that led to persistent cognitive deficits
- Use of neuroleptics
- Use of celiprolol and sotalol
- Use of doses of corticosteroids that may interfere with the pharmacodynamic measurements performed in the study
- Use of Monoamine oxidase A/B inhibitors
- Current use of centrally acting anticholinergics
- Use of benzodiazepine within 48 hours before a study day
- Current use of a cholinesterase inhibitor
- Alcohol abuse (defined as use of alcohol despite significant areas of dysfunction, evidence of physical dependence, and/or related hardship due to alcohol)
- Use of recreational drugs
- Concomitant use of inhibitors of CYP2D6 or of CYP3A4 (unless on a stable dose without any recent or upcoming changes)
- Any other condition that in the opinion of the investigator would complicate or compromise the study or the wellbeing of the subject
- Any contraindication for magnetic resonance imaging

### Procedures

**Figure 1:**
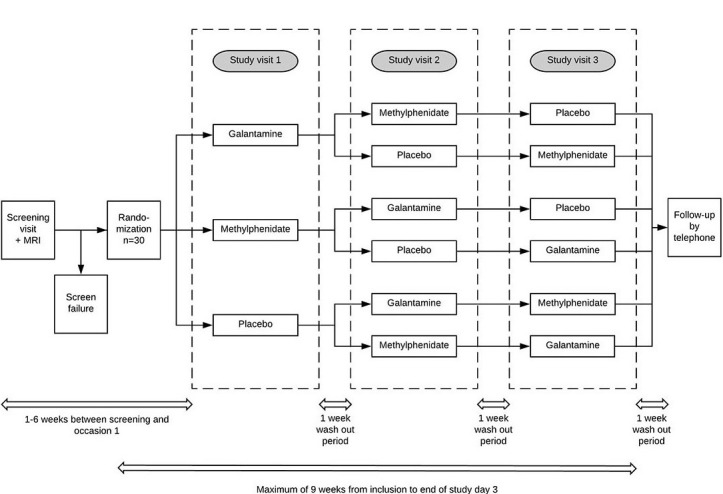
Schedule diagram of the study. During the screening visit, a magnetic resonance imaging (MRI) is performed. After randomization, a person is placed in one of the 6 study arms. One week after the last study visit, follow-up by telephone will take place. Between each study visit, a wash-out period of one week is scheduled.

Prior to any study-related procedures, written informed consent for the study was obtained from each subject. The study consisted of a screening visit, followed by three study visits (challenge phase). The screening visit occurred at approximately 1 to 6 weeks prior to the first study visit. After inclusion, the study lasted a maximum of 9 weeks until the end of the third study visit. Between each study visit, a wash-out period of at least one week was scheduled. About 7 days after the end of the third study visit, participants were contacted by telephone to inquire about possible side effects. An overview of the study can be seen in Figure 1.

#### Screening Visit

A full medical screening (medical history, physical examination, vital signs in supine position, 12-lead electrocardiogram (ECG), urinalysis and routine hematology, biochemistry and electrolytes) was performed to assess a subject’s eligibility for this study and to assess possible safety concerns of administrating the study medication. Extensive information of the medical screening can be found in Multimedia Appendix 1. All participants were thoroughly trained and familiarized with the central nervous system (CNS) tests on the screening visit in order to minimize learning effects during the study. The tests were performed in a quiet room with ambient illumination with only 1 participant in the room per session. When a person met the criteria for inclusion, an MRI was performed on the same day. An overview of the screening visit can be seen in Table 1.

#### Study Visit

On each study visit, safety measures were performed prior to drug administration, consisting of vital signs, 12-lead ECG and urinalyses. Vital signs were checked again halfway through the visit and at the end of the occasion. During a study visit, 5 rounds of CNS tests were performed. Table 1 shows an overview of the assessments during a study visit.

### Intervention

During three separate study visits, subjects received a single pharmacological challenge with galantamine, methylphenidate, or placebo in a random order. The Department of Clinical Pharmacology and Pharmacy of the VUmc manufactured galantamine capsules, methylphenidate capsules, and its matching placebo for oral use and guarded stability of the products.

#### Galantamine

In this trial, a dose of 16 mg was administered (2 tablets of 8 mg). Galantamine is a reversible competitive inhibitor of acetylcholinesterase and also has activity as an allosteric modulator of nicotinic acetylcholine receptors [14]. In several randomized, double-blind, placebo controlled clinical trials, galantamine was effective in people with probable Alzheimer’s disease [9-13]. The usual starting dose of galantamine treatment is 8 mg per day [26]. In a previous study the dose was upgraded to 16 mg per day to objectify a good clinical effect [27-29]. Adverse events of galantamine are particularly cholinergically mediated events affecting the gastrointestinal system such as nausea and vomiting which occur in >10% of the people [26].

#### Methylphenidate

Methylphenidate (MPH) is an indirectly working sympathicomimetic drug with effects comparable to amphetamines and a potent dopamine, norepinephrine, and serotonin releaser that also inhibits the uptake of the released biogenic amines into presynaptic neurons [15-17]. The dose of MPH was chosen at 10 mg (2 tablets of 5 mg), taken orally. This dose and administration of MPH was chosen based on strategies used in previous trials in the elderly depressed, open-label administration guidelines in the demented population and because a preliminary study of MPH for apathy provided data on the safety and efficacy of 10 mg of methylphenidate administered two times a day [30]. The main adverse effects of MPH are agitation, sleep problems, reduced appetite, and palpitations. MPH is also associated with a modest rise in blood pressure and heart rate [31].

### Measures

#### Pharmacodynamic Assessments

A series of CNS tests were administered using the ‘NeuroCart’ to study the acute effects of the intervention on a set of the CNS drug responsive domains (Table 2) [32].

**Table 1:** Overview of the screening and the study visits. On the study days, the pharmacological challenge is administered at time point 0.

Assessments		Screening visit	Study visits (1, 2, 3) with time points (hours)							
			-1.5	0	1.0	1.5	2.0	2.5	3.0	3.5
**Clinical measures**										
	Clinical procedures^a^	X^b^								
	Vital signs	X	X			X				X
	12-lead electrocardiogram	X	X							
	Urinalysis	X	X							
	Clinical laboratory^c^	X								
	Pharmacokinetics blood sampling		X		X			X		X
	Drug administration			X						
**Central nervous system tests**										
	Eye movements	X	X		X			X		X
	Adaptive tracker	X	X		X			X		X
	Visual Analog Scales	X	X		X			X		X
	Pharmaco-electroencephalography		X		X			X		X
	Visual Verbal Learning Test-15	X					X	X	X	
	Facial recognition task	X						X		
	N-back task	X	X		X			X		X
	Stop Signal test	X	X		X			X		X
Magnetic resonance imaging		X								

^a^Clinical procedures include medical history and medication use, Mini-Mental State Examination, Clinical Dementia Rating scale, and physical examination.

^b^X: the assessment was performed.

^c^Clinical laboratory includes hematology and blood biochemistry.

**Table 2:** Functions measured by each task.

Task	Function				
	Executive Functioning	Memory	Psycho­motor speed	Vigilance	Subjective Drug Effects
N-back task	X^a^	X			
Stop Signal Task	X				
Adaptive tracking	X		X	X	
Visual Verbal Learning Test–15		X			
Facial encoding and recognition task		X			
Eye movements			X		
Pharmaco-electroencephalography			X		
Bond and Lader Visual Analog Scale					X

^a^X: task belongs to the function in the column.

Executive functioning was measured by the tasks adaptive tracking [33-35], Stop Signal Task [36,37], and N-back task [38-40]. Memory was assessed by Visual Verbal Learning Test-15 (VVLT-15) [41], N-back task and the Facial Encoding and Recognition Task [38,42]. The VVLT-15 contains 3 different subtests. The immediate word recall test was performed first; after an interval of approximately 60 minutes, the delayed word recall test and then the delayed word recognition test were performed. Our main outcome is defined as the change in performance after a pharmacological challenge on the VVLT-15 and the adaptive tracker.

Besides executive functioning and memory the following functions were measured: psychomotor speed, vigilance, and subjective drug effects. The following Neurocart tests were used for the measurement of these functions: saccadic and smooth pursuit eye movements [33,34], adaptive tracking, and Bond and Lader Visual Analog Scale [43,44]. In Table 2, the tests with corresponding cognitive functions can be seen.

#### MRI measurements

The MRI was acquired on 3T whole-body MR system (Discovery; GE Medical Systems Milwaukee, WI, USA), using an eight-channel head coil at the VUmc. The following sequences were applied: T1-weighted imaging, T2-weighted imaging, fluid-attenuated inversion recovery (FLAIR), Diffusion Tensor Imaging (DTI) MRI and resting-state functional MRI (rs-fMRI). In total, the imaging took about 40 minutes. There was no intravenous contrast administration. All scans were checked by a neuroradiologist for unexpected gross abnormalities.

Medial temporal lobe atrophy was rated on the coronal reconstructions of the T1-weighted images with scores ranging from 0-4 [45]. Posterior atrophy was rated on the combination of T1-weighted and FLAIR sequences and global cortical atrophy was rated on FLAIR sequences using a 4-point visual rating scale (0–3) [46-48].

White matter hyperintensities (WMH) were rated using the Fazekas scale, with scores ranging from 0-3, on the FLAIR images [49]. Microbleeds were defined as small round hypointense foci on T2*-weighted images, with a maximum diameter of 10 mm located in brain parenchyma. Lacunes were defined as deep lesions (3-15 mm) with cerebral spinal fluid—like signal on all sequences. A rater who was blinded for all clinical information assessed the scores.

We are currently preprocessing all images. They will be normalized to standard Montreal Neurological Institute (MNI) space with FSL software [50,51]. Presence and location of lacunar infarcts in each subject will be assessed and the severity and location of WMH will also be measured using automated segmentation [52]. Structural connectivity will be assessed using DTI “fiber tracking” with FSL software. We will segment specific white matter tracts part of the cholinergic and monoaminergic systems, by means of probabilistic tractography. Diffusion properties (fractional anisotropy and mean diffusivity) will be investigated along the tract pathways. Functional connectivity will be assessed using rs-fMRI. Individual connectivity maps will be identified using standard resting state network maps from FSL. These maps include eight resting state networks, including the DMN and executive control network.

#### Pharmacokinetic assessments

Blood samples (4mL) for plasma concentrations of galantamine and methylphenidate were collected repeatedly. According to protocol, blood samples were taken before administration of the medication and 1 hour, 2.5 hours and 3.5 hours after administration (Table 1). The exact dates and times of blood sampling were recorded. Samples were centrifuged at 2000G during 5 minutes at 4 degrees Celsius. Plasma was transferred into 2 mL Sarstedt tubes by pipette. The plasma was stored at -20 degrees Celsius for the most optimal stability until analyses. For the analysis of galantamine and methylphenidate, two dedicated liquid chromatography—mass spectrometry / mass spectrometry methods were developed. Each method was specific and sensitive for the analysis of interest. Bioanalysis was performed by the Pharmacy at the VUmc, Amsterdam. ADAPT II Release 4 software was used [53]. Pharmacokinetic parameters will be estimated using compartmental analysis.

### Statistical analyses

#### Sample size

Based on a recently performed study at the Centre for Human Drug Research in people with Alzheimer’s disease, acute effects of galantamine on Neurocart tests have been measured (CHDR0915). In this study, the difference in adaptive tracking performance between galantamine and placebo occasions was 2.07% with a standard deviation of 3.35. Assuming that a comparable efficacy can be seen in people with VCI and monoaminergic neuronal dysfunction supplemented with methylphenidate, and assuming a similar standard deviation, we would need at least 24 subjects with VCI to show a mean difference of approximately 2.0% (on adaptive tracker) with a power of 80%. For the galantamine challenge, the VVLT-15 was used to calculate the sample size. In this study, the difference in VVLT-15 between galantamine and placebo was 3 words with a standard deviation of 3.0. Assuming that in people with VCI and cholinergic neuronal dysfunction supplemented with galantamine, a 2-word difference should be possible, and assuming a similar standard deviation, we would need at least 24 people with VCI. Taking into account a 25% drop out rate, we enrolled 30 subjects.

#### Planned analyses: Pharmacodynamics

The main outcome of this study is the individual change on the CNS tests after a pharmacological challenge. Participants will be categorized as a responder or nonresponder (defined as a significant difference on Neurocart tests) on both active conditions. Statistical analyses of outcome measures will be performed by using mixed-model analyses of variance (ANOVA) with treatment, period, time, and treatment by time as fixed factors; participant, participant by treatment, and participant by time as random factors; and the average baseline measurement as covariate for each test on each time point. Single-measured parameters without pre-value measurement will be analyzed with a mixed-model ANOVA with treatment and period as fixed factors and subject as random factor. The Kenward-Roger approximation will be used to estimate denominator degrees of freedom and model parameters will be estimated using the restricted maximum likelihood method. The general treatment effect and specific contrasts are reported with the estimated difference and the 95% confidence interval, the least square mean estimates, and the *P* value. For repeated measures, graphs of the Least Squares Means estimates over time by treatment will be presented with 95% confidence intervals as error bars, as well as change from baseline Least Square Means estimates. All statistical hypothesis tests are conducted at alpha=0.05 (two-sided). No adjustments for multiple comparisons will be applied.

Furthermore, we will correlate the location and severity of the cerebrovascular lesions to the derived white matter fiber tracts and neuronal networks. ANOVAs adjusted for age, sex, and baseline cognition will be performed to associate MRI measures for structural and functional connectivity with a positive response to the cholinergic and to monoaminergic challenge. Subsequently, we will use logistic regression to identify the most optimal combination of MRI measures to predict response.

#### Planned Analyses: Pharmacokinetics

Where appropriate and possible, the relationship between plasma concentrations of galantamine/methylphenidate and a corresponding selection of relevant pharmacodynamic measurements will be defined and the data will be plotted to evaluate the relationship graphically. If deemed appropriate and possible, a suitable pharmacokinetic/pharmacodynamic model may be applied to describe the exposure/concentration-effect relationship.

### Ethical Considerations

The protocol of this study was approved by the Medical Ethics Committee of VU University Medical Center (protocol number 2013.393) and the competent authority (number NL45933.029.13). The trial is registered at the European Union Clinical Trials Register (2013-003396-35). The study was conducted according to the Dutch Act on Medical Research involving Human Subjects. An independent monitor (quality manager) of the Centre of Human Drug Research monitored the study data according to Good Clinical Practice.

All adverse events reported spontaneously by the subject or observed by the investigator or his/her staff were recorded. The occurrence of an adverse experience that was fatal, life-threatening, disabling, required or prolonged in-patient hospitalization, or caused congenital anomaly was described as a serious adverse event (SAE). A Suspected Unexpected Serious Adverse Reaction (SUSAR) was defined as an unexpected serious adverse reaction in subjects given a drug that may or may not have been dose related, but was unexpected, as they were not consistent with current information.

## Results

The first participant was enrolled in March 2014. Participant enrolment was completed in September 2017. After data-cleaning and locking of the database, we will start analyzing the results. We expect to publish the results in 2018. No SAEs or SUSARs have occurred during the study.

## Discussion

The STREAM-VCI is a double-blind, three-way, case cross-over trial, in which people with VCI received methylphenidate, galantamine, or placebo in a random order. With this trial, we aim to improve executive function and memory in people with VCI using pharmacological interventions aimed at the enhancement of monoaminergic and cholinergic neurotransmitter systems.

VCI is one of the most important subtypes of cognitive impairment [1,2,54,55], and as of date, there is no approved symptomatic treatment for people with VCI. Evidence suggests that executive dysfunction and memory complaints in VCI are caused by damage to monoaminergic and cholinergic neurotransmitter systems, respectively [8,20-24]. In the past, several studies have investigated the effect of pharmacological intervention with methylphenidate and a cholinesterase inhibiter. However, the results of these studies were contradictive with some studies showing a positive effect of the intervention and other studies showing no effect [16,19,56]. A possible explanation for the lack of conclusive results may be the heterogeneity in symptoms displayed by people with VCI. Previous studies did not take this interpatient variability into account. By using structural and functional connectivity measures of the cholinergic and monoaminergic tracts, we aim to visualize how vascular damage affect these tracts in each person. By doing so, we aim to understand why some people respond to the challenge and some people do not. In this proof-of-concept study, we expect that people with VCI with executive dysfunction due to vascular damage to the monoaminergic neurotransmitter system will respond to a monoaminergic challenge and that people with VCI with memory dysfunction caused by vascular damage to the cholinergic system will have a positive response to a cholinergic challenge.

STREAM-VCI is the first study to take the heterogeneity of people with VCI into account by correlating the cognitive symptoms with structural and functional connectivity in monoaminergic and cholinergic systems measured with structural and functional MRI and by correlating these changes with a positive response to a challenge with galantamine or methylphenidate. Based on this information, we aim to develop a prediction model that estimates a positive response to a cholinergic and/or monoaminergic challenge in people with VCI. This could be a major step forward towards individually-tailored pharmacological interventions aimed at the affected neurotransmitter systems.

## Appendix

Multimedia Appendix 1 Safety measurements on screening day.

## Abbreviations

ANOVA: analyses of variance
CNS: central nervous system
DMN: default mode network
DTI: Diffusion Tensor Imaging
ECG: electrocardiogram
FLAIR: fluid-attenuated inversion recovery
MMSE: Mini-Mental State Examination
MNI: Montreal Neurological Institute
MPH: Methylphenidate
MRI: magnetic resonance imaging
rs-fMRI: resting-state functional magnetic resonance imaging
SAE: serious adverse event
STREAM-VCI: Symptomatic Treatment of Vascular Cognitive Impairment
SUSAR: Suspected Unexpected Serious Adverse Reaction
VCI: vascular cognitive impairment
VUmc: VU University Medical Center
VVLT-15: Visual Verbal Learning Test-15
WMH: white matter hyperintensities
