# Supplementary material for: Symptomatic Treatment of Vascular Cognitive Impairment (STREAM-VCI): Protocol for a Cross-Over Trial
Source: JMIR Res Protoc. 2018 Mar 20;7(3):e80. doi: 10.2196/resprot.9192 (PMC5883073; doi:10.2196/resprot.9192)
Supplement: Multimedia Appendix 1 [file resprot_v7i3e80_app1.pdf]

Appendix 1. Safety measures on screening day

Safety clinical tests

Blood pressure

Automated oscillometric blood pressures were measured using a Nihon-Kohden BSM-1101K monitor or a Colin Pressmate BP 8800. Additionally, the heart rate data using BSM-1101K monitor was provided by the pulse oximeter attached to the monitor. Two measurements were performed and the last reading was taken as the value to be recorded. Vital signs were taken at the screening visit.

Electrocardiogram

An ECG was obtained at screening using Marquette 800/5000/5500 The ECG was reviewed by a physician. The following parameters were reported: PR-, QRS-, QT-, QTc-interval and heart rate.

Safety laboratory tests

Hematology

At screening, a sample of 4 ml of venous blood was collected in a vacutainer on EDTA. Samples were analyzed by the Central Clinical Hematology Laboratory of VU University Medical Center. The following assessments were performed: Hemoglobin, hematocrit, red cell count (RBC), total white cell count, mean cell volume, leukocyte differential count and platelet count, BSE, CRP.

Blood Biochemistry

At screening, in total 21 mL of venous blood was collected in a SST® Gel and Clot Activator tube(Becton and Dickinson), EDTA tube and heparin tube. Samples were analyzed by the Central Clinical Chemistry Laboratory of VU University Medical Center. The following assessments were performed: Sodium, potassium, calcium, inorganic phosphate, total protein, albumin, (sober) glucose, (sober) total cholesterol, triglycerides, HDL-cholesterol, LDL-cholesterol, (sober) homocysteine, blood urea nitrogen, creatinine, uric acid, total bilirubin, alkaline phosphatase, AST, ALT, gamma-GT and LDH, TSH, FT4, vitamin B1, vitamin B6, vitamin B12 and folic acid.

Urinalysis

At screening, routine urinalysis was performed by dipstick (Multistix 10 SG® (Bayer, Mijdrecht, The Netherlands) for the following parameters: leucocytes, blood, nitrite, protein, urobilinogen, bilirubin, pH, specific gravity, ketones, and glucose. Abnormal results waere subsequently microscopically analyzed by the Central Clinical Chemistry Laboratory of VUmc.
